# What Contributes to the Regularity of Patients with Hypertension or Diabetes Seeking Health Services? A Pilot Follow-Up, Observational Study in Two Sites in Hubei Province, China

**DOI:** 10.3390/ijerph13121268

**Published:** 2016-12-21

**Authors:** Da Feng, Ray Serrano, Ting Ye, Shangfeng Tang, Lei Duan, Yuan Xu, Jian Yang, Yuan Liang, Shanquan Chen, Zhanchun Feng, Liang Zhang

**Affiliations:** 1 School of Medicine and Health Management, Tongji Medical College, Hua Zhong University of Science and Technology, No.13 of Hangkong Road, Qiaokou District, Wuhan 430030, China; fdnunu@163.com (D.F.); yeting@hust.edu.cn (T.Y.); sftang@hust.edu.cn (S.T.); dlhust@sina.com (L.D.); yuanhu_hsu@126.com (Y.X.); yangjian1228@hust.edu.cn (J.Y.); zcfeng@mails.tjmu.edu.cn (Z.F.); 2 Department of Health Policy and Management, Rollins School of Public Health, Emory University, Atlanta, GA 30322, USA; ray.serrano@stanfordalumni.org; 3 School of Public Health, Tongji Medical College, Hua Zhong University of Science and Technology, No.13 of Hangkong Road, Qiaokou District, Wuhan 430030, China; liangyuan217@163.com; 4 Jockey Club School of Public Health and Primary Care, Chinese University of Hong Kong, Hong Kong, China; shanquan0301@gmail.com

**Keywords:** hypertension, diabetes, regular, health seeking behavior, usual provider, social factors

## Abstract

Regular maintenance of non-communicable chronic diseases can constrain disease progression in diabetic and hypertensive patients. To identify the individual and social factors that are associated with positive health-seeking behaviors and regular maintenance of chronic diseases, we have conducted a follow up study in 2015 on diabetic and hypertensive patients in Hubei Province. We used binary logistic regression models to determine specific factors associated with diabetic and hypertensive patients that sought healthcare services for their conditions in accordance with current Chinese Centers for Disease Prevention and Control (CDC) guidelines. Our findings show that 42.16% of 510 people living with chronic conditions (PLCDs) sought health services in line with existing guidelines. Findings also show a higher probability (8.418 times) for PLCDs seeking healthcare services at higher-tiered hospitals (secondary and tertiary hospitals) than for PLCDs seeking care at primary hospitals (odds ratio (OR) = 8.418, 95% confidence interval (CI) = 4.82, 14.27, *p* < 0.001). These analyses underscore the importance of having patient advocates who can provide support, where necessary, and encourage positive health-seeking behavior. The study also shows a negative impact on regular maintenance for PLCDs in households with high financial constraints. In contrast, the study shows positive impacts for increased household income, age, and residency in rural locations. In sum, this study underscores the importance of primary hospitals as key points of care and critical players in care coordination for PLCDs. The study provides more evidence for Chinese policymakers seeking to contain costs and improve population health. The findings also underscore the need for community-based interventions, specifically interventions that link local primary hospitals, friends/family members, and PLCDs.

## 1. Introduction

Non-communicable chronic diseases are major sources of mortality throughout the world [1]. In 2012, approximately 38 million people living with chronic conditions (PLCDs) succumbed to their conditions and more than 40 percent of these deaths were premature. Roughly half of PLCDs who died in 2012 were under 70 years of age. The same challenge is found worldwide. Nearly three-quarters of all deaths from chronic disease occurred in low- and middle-income countries [1]. 

In China, the Fifth Chinese National Health Services Survey, conducted in 2013, reported that approximately 270 million Chinese people have been diagnosed with at least one chronic condition. The Survey also noted that nearly 82% of deaths and 70% of disability-adjusted life years were lost due to chronic conditions [2]. A recent study predicted that by 2020, the number of deaths attributed to chronic conditions will rise to 85% [3]. At the same time, several studies suggest that the majority of premature deaths caused by chronic conditions were largely preventable [4,5,6]. Hypertension and diabetes have the highest incidence among all chronic diseases in China in addition to being considered major sources of mortality and morbidity [7,8]. 

The rising prevalence of hypertension and diabetes in recent decades has drawn the attention of Chinese policymakers. To enable any set of policies targeting chronic conditions, policymakers must work within China’s three-tiered healthcare delivery systems. In rural areas, healthcare is delivered via a loose network of village clinics, township hospitals, and county hospitals. Similarly, in urban centers, community health centers and city hospitals service the population by providing diagnostic and treatment services. The Chinese Centers for Disease Prevention and Control (CDC) have recently sought to integrate efforts from various hospital tiers in efforts to mitigate the impact of chronic diseases like hypertension and diabetes. The New Health Care System Reform, launched in 2009, made the management of chronic diseases a critical element of publicly-delivered healthcare services. It also covered services for all diabetic and hypertensive patients free of charge. These services include glycemic and blood pressure measurement, patient-centered instructions for medications, and health education services [9,10]. To date, there is substantial evidence to suggest that regular health services utilization is significantly associated with long-term glycemic and blood pressure control in addition to showing that regular preventive care and guidance can reduce diabetes-related complications and the reoccurrence of hypertension [11,12,13]. Self-management such as regular self-monitoring of blood glucose and blood pressure and healthy lifestyle choices [14,15] were also shown to reduce the impact of hypertension and diabetes. 

When PLCDs did not seek care and maintain their conditions in line with Chinese CDC recommendations, self-management strategies for hypertension and diabetes were poorly monitored and often insufficient. One policy solution was to systematize information and communication channels so that patient self-monitoring devices (e.g., glucose monitoring devices and automated blood pressure devices) could inform and motivate PLCDs to actively seek healthcare services and self-manage their conditions in line with CDC recommendations [16]. Unfortunately, in spite of many efforts to promote self-monitoring devices for PLCDs, the devices remain underutilized, particularly among underserved populations. As a result, PLCDs were less likely to be exposed to regular self-care reminders that are consistent with up-to-date CDC guidelines [17,18]. 

In this study, we have examined the individual and social factors that are associated with regular self-maintenance and positive health-seeking behaviors for PLCDs. Our analyses address these factors in a variety of areas. To address social factors, for example, we have examined the role that a household head (i.e., the principal economic contributor) plays in the health status and health-seeking behaviors of household members. Moreover, we have studied urban–rural economic differences to show the importance of macro socio-economic factors. We also have evaluated the role of social capital and social support such as patient advocates, specifically family and friends, who may encourage patients to regularly seek preventive healthcare services.

To address individual characteristics, we have examined the role of age, income, and level of chronic disease in positive health-seeking behaviors among PLCD. Our analyses in these areas are in line with existing literature [19,20]. For example, there is evidence to support the notion that elderly who live alone, are ambulatory, and have high self-reported health status prefer fewer clinical visits suggesting that less—not more—utilization of healthcare services may actually coincide with improved health outcomes for PLCD. With respect to income, patients who report high income and lower economic burden of disease also positively reported having access to needed health services [21,22,23]. To date, however, few studies have explored the determinants of health-seeking behavior for PLCDs from a social perspective. These determinants could include social support/hospital accompaniment (e.g., whether a PLCD has someone to accompany them when go to the hospital) and primary hospitals (e.g., village clinic; community or township hospitals) as the usual source of care. In sum, current evidence on health-seeking behaviors and positive self-maintenance of chronic conditions for PLCD in China have focused too narrowly on the patterns of health service utilization [24] and willingness to use health services [3,9]. There is, however, limited understanding the individual- and social-level factors that are associated with regular utilization of healthcare services by PLCD. This study sought to address the dearth in existing literature and examined the individual- and social factors that underpin positive health-seeking behavior. We hope that our findings will ultimately help inform interventions that promote healthy-living and regular utilization of preventative care services among Chinese PLCD. 

## 2. Materials and Methods 

### 2.1. Study Design 

**Figure 1:**
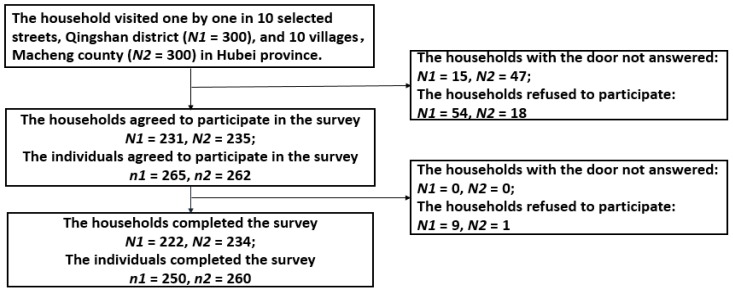
Recruitment of Survey Participants. Note: N1: household number in Qingshan; N2: household number in Macheng; n1: individual number in Qiangshan; n2: individual number in Macheng.

The study was conducted in 2015. To obtain data, we employed experienced general practitioners from the local grassroots medical institutions (township hospitals or community centers) to be interviewers. We instructed these practitioners to not render any services during the interview sessions. We have focused on Macheng County to examine PLCDs residing in rural areas and in Qingshan District, we have examined PLCD residing in urban settings. Both study areas are located in Hubei Province, which was also the location for many of the samples for the 2013 Fifth National Health Service Survey of China in 2013. In total, five surveyors were invited from every grassroots medical institution and a total of 48 surveyors were employed to conduct these investigations. Using a stratified multi-stage sampling method, we have randomly selected five townships or sub-districts, and ten villages or communities from each county or district. The households with at least one diabetic or hypertensive member were eligible for this study. According to health records which were retrieved from the essential public health services program, all diabetic and hypertensive patients from selected villages or communities were located and added to a comprehensive list. These households were then divided into three groups according to their distance to the nearest healthcare facility (e.g., <1 km, 1 to 2 km, and >2 km), and 10 samples were randomly selected from each group. Finally, three hundred households from rural areas and 300 households from urban areas were included (Figure 1).

Face-to-face interviews of households were conducted at baseline. Written consent was obtained from participants and data on personal contact information, socio-demographics, chronic disease of family members, and household income were also collected. Periodic telephone interviews were conducted at every month for one year to obtain information on patients’ utilization of health services. Recruitment and response rates are shown in Figure 1. In total, 600 households were visited, 62 of them did not answer and 82 of them refused to participate due to the death of the PLCD (1.6%), migration (4.3%), and survey fatigue (56.0%), and miscellaneous reasons (38.1%) during this study. In the end, 510 individuals from 456 households (e.g., 222 households from Qingshan District, 234 of them from Macheng County) completed the entire series of interviews. 

### 2.2. Measurements 

In 2013, new guidelines were set by the Chinese CDC to guide the management of diabetic and hypertensive patients at different grades. All PLCD at grade 3 (i.e., most severe level), grade 2 (i.e., moderate level), and grade 1 (i.e., mild level) were advised to seek health services regularly (every months for grade 3, every two months for grade 2, and every three months for grade 1) [25,26,27]. 

Based on available health records, we have stratified our samples of diabetic and hypertensive patients according to severity—a determination made by physicians through clinical diagnoses made prior to the study. According to the new guidelines, regular health-seeking behavior was coded as a binary dependent variable, which indicated whether each PLCD regularly sought health services. We then measured their responses via a continuous series of follow-up interviews in 2015. If a PLCD demonstrated a pattern of health seeking behavior consistent with CDC guidelines for their chronic disease grades, they were coded as “regular” in our study. And if the frequency of seeking health services was lower or and inconsistent with CDC guidelines, we coded them as “irregular”.

Independent variables were individual factors that included: (1) Age (<50, 50–59, 60–69, and 70 years and above) [23]; (2) education level (no formal education, 1–6 years study, 7–9 years study, and >9 years study) [22]; (3) comorbidity (i.e., specific types of chronic diseases) [28,29]; and (4) the self-reported health score ranging from 0 to 100 was measured by asking which number reflected their health status well [30]. The groups of below 60, 60–80, and above 80 were divided and defined as poor-, fair-, and good- status, respectively. 

Economic factors were also examined. They include: (5) heavy economic burden of disease, which was defined by a household out-of-pocket health expenditure that reached or exceeded 40 percent of disposable household income in the past year (WHO Catastrophic Health Expenditure) [28,29]. Expenditures on medical care and total household expenditures were estimated through the self-reported consumption in the past 30 days during our telephone interviews. We calculated these expenditures as a percent of annual medical expenditure accounting for total household income, and adjusted to normal and high normal groups based on the previously cited criterion; (6) Household income in this study was interpreted as urban household disposable income and rural household net income. Household income for the previous year was stratified in two ways using the 2015 criteria cited in the Hubei Statistical Yearbook. For rural residents, we data stratified as: low level (less than CNY 20,000), fair level (CNY 20,000 to CNY 40,000), and medium level (CNY 40,000 or above). For urban areas, we stratified by: low level (less than CNY 30,000), fair level (CNY 30,000–60,000), and good level (CNY 60,000 or above) [30]; (7) The type of health insurance consisted of medical insurance for urban workers, medical insurance for urban residents, and the New Rural Cooperative Medical System (NRCMS). 

The social factors sought to examine the impact on PLCDs who: (1) were the head of household (yes, no); (2) resided in a rural or urban setting; (3) had social support (i.e., someone to accompany him/her to clinical visits); (4) Also, we accounted for the number of household members residing with PLCD who also have chronic diseases that are grades 1, 2, or above; (5) to determine usual source of care, we asked participants about the types of hospitals they usually chose to visit when they are sick or in need of medical advice [31]. Responses were coded according to the three levels of hospitals, i.e., village or community clinics belonged to first level, township or community hospitals belonged to second level, and county hospitals/municipal level-or-above hospitals are belonged to the third level; (6) Contribution to household income was coded in three ways: primary source (50% or above), secondary source (less than 50%), and no financial contribution [32].

### 2.3. Statistical Analysis 

Descriptive analyses were carried out for socio-demographic characteristics of all participants. Prior to conducting binary logistic regression models to identify the factors associated with positive health-seeking behavior among PLCD, a series of Chi-square tests were used to explore differences in covariate values between regular and irregular groups. From these tests, we identified individual and social factors that could be included in our regression analyses. We then applied a binary logistic regression analyses with forward stepwise to examine potential predictors of regular health service utilization. Logistic regression coefficients were estimated and tested by the Wald statistic. Lastly, we calculated adjusted odds ratios (OR) and 95% confidence intervals (CIs) and for all comparisons, two-sided *p*-values < 0.05 were considered statistically significant. All statistical procedures were performed using the Statistical Package for Social Sciences v 22.0 (SPSS Inc., Chicago, IL, USA). 

### 2.4. Ethical Statement

This research was approved by the ethics committee of Tongji Medical College, Huazhong University of Science and Technology. The ethical code was No: IORG0003571. Informed consent documents and survey questionnaires were thoroughly reviewed and approved.

## 3. Results

### 3.1. Socio-Demographic Characteristics of Participants 

Patients were predominantly aged above 60 (66.5 percent) and had a mean age of 64.60 years. Slightly more than half of the participants were female (50.8 percent) and had an above middle school education level (51.57 percent). Participants mostly belonged to the households of two or more members (94 percent), and the majority had hypertension (78.45 percent). Approximately 36.5 percent participants are at grade-1, 39.8 percent participants are at grade-2, 23.7 percent participants are at grade-3. More details are presented in Table 1. 

### 3.2. Factors Affecting Regularity of Health Seeking Behavior among Patients with Hypertension or Diabetes 

Among 510 participants, results of bivariate analyses indicated significant differences between regular and irregular groups with respect to several variables. Regular health seekers tended to be elderly, have lower economic burden of chronic diseases, have higher household income, have medical insurance for urban workers, have someone to accompany them to hospital visits, live with more than one type of chronic disease, and reside in rural areas. No significant differences were identified between regular and irregular health seekers across the subgroups of self-reported health score, educational level, household size, and economic contribution to the family (Table 2).

### 3.3. Predictors of Regular Health-Seeking Behavior among Diabetic and Hypertensive Patients 

There were bivariate effects of predictors on the dependent variable, which could be confounded by other factors. Consequently, we used a multivariate binary logistic regression analysis to examine the effect of each potential predictor identified in the bivariate analysis. Except for self-reported health scores, education, household size, and economic contribution to the family, all variables were tested for association with regular vs. irregular health-seeking behavior using a multivariate logistic regression. The details are shown in Table 3.

Social factors—including usual provider, social support, urban/rural residence—appear to have contributed the most to regular health services utilization by PLCD. The odds of a PLCD who regularly sought health services from higher-tiered hospitals was 8.418 times greater than for PLCDs who regularly sought health services at primary hospitals (OR = 8.418, 95% CI = 4.82, 14.27, *p* < 0.001). Similarly, the presence of social support (e.g., a family member or friend to accompany one to hospital visits) was highly associated with the PLCDs who regularly sough healthcare services (OR = 1.598, 95% CI = 2.813, 8.691, *p* < 0.001). Moreover, regular healthcare seekers residing in rural areas were 2.837 times more likely to be categorized as ‘regular’ than those residing in urban areas (OR = 2.387, 95% CI = 1.352, 4.212, *p* = 0.003). 

Economic factors including household income and economic burden of chronic diseases were also associated with the regular healthcare-seeking behavior. Compared with the families whose annual household income was at low levels, participants living with families whose household income was at fair or high levels seemed more likely to regularly seek healthcare services (OR = 6.031, 95% CI = 3.153, 11.535, *p* < 0.001; OR = 6.097, 95% CI = 3.341, 11.126, *p* < 0.001). Meanwhile, the heavy economic burden of chronic diseases was inversely related to regular health-seeking behavior by PLCD (OR = 5.611, 95% CI = 3.273, 9.618, *p* < 0.001).

Age was the only socio-demographic variable that was significantly associated with regular healthcare-seeking behavior. Compared with patients aged below 50, patients with higher ages sought health services more regularly and in accordance with CDC guidelines. Lastly, when compared to having one chronic disease, PLCDs suffering from more than one chronic diseases were more likely to report regular health-seeking behavior. PLCDs who suffered from more than two sorts of chronic diseases were 4.473 times more likely than PLCDs suffering from one chronic disease to report regular health-seeking behavior (OR = 4.473, 95% CI = 1.843, 10.855, *p* = 0.001).

## 4. Discussion 

In this study, we have explored factors that influence regular healthcare utilization (i.e., utilization that is consistent with Chinese CDC guidelines) by diabetic and hypertensive patients in Hubei Province, China. Our findings show that social factors such as the higher-tiered hospitals and support by family/friends were significantly associated with regular healthcare-seeking behavior. Throughout our 12-months study, we have found that patients preferred higher-tiered hospitals as their usual source of care, but also reported regular healthcare-seeking behavior more frequently than PLCDs seeking care at primary hospitals. This finding may be explained by the common public perception that healthcare services provided by higher-tiered hospitals are more trustworthy and accessible, and as a result, may be more responsive to the needs of PLCDs [33,34]. Current evidence posits that having a usual source of care will translate into a trustful and satisfactory relationships between PLCDs and their providers [35]. A recent community-based study conducted in a southern region in China demonstrated that patients with chronic conditions tended to use secondary care instead of primary care as their regular source of care [36]. Our findings are consistent with this line of evidence. We have found that higher-tiered hospitals attracted most PLCDs in addition to playing a critical role in regular healthcare-seeking behavior. At the same time, however, we acknowledge that this finding may weaken the long-term effect of regular healthcare-seeking behavior on overall maintenance of diabetes and hypertension. On the one hand, the inefficiencies stemming from the overutilization of high quality resources may be incurred. These inefficiencies may cause overcrowding and diminished care for more severe cases [37]. On the other hand, a lack of health information exchange systems or mechanism across vertical three-level healthcare service networks might be another important factor for chronic disease management for PLCDs.

Approximately 75.35% of the regular healthcare seekers chose higher-tiered hospitals, a finding that underscores persistent gaps in access to high-quality healthcare services. The finding is no different across China where primary hospitals have historically remained underutilized. At present, chronic disease management is a core component of essential public health services covered for all patients with diabetes and hypertension. These services are provided free of charge and are monitored via up-to-date patient health records [33]. In spite of systematic efforts to improve regular health-seeking behavior by PLCD, our findings suggest that PLCDs remain hesitate to seek care at primary hospitals. These findings are in line with existing evidence that suggests that most patients are unwilling to seek health services from primary hospitals because of long wait times, limited selection of medications, and low-quality health services. If anything, our study further underscores the importance of improving the ability and services available at primary hospitals to effectively meet the needs of PLCDs. We posit that this goal could be reached through health promotion campaigns to improve regular healthcare utilization by PLCDs and through strategic use of health information exchange systems across vertical networks. Combined, these two suggestions could link PLCDs to timely and high-quality care at primary hospitals [37].

Social support is significantly associated with positive health-seeking behavior and regular self-maintenance [35,38]. Our study’s inclusion of family/friend support addressed the key role of social capital and social support. Findings suggesting a strong association between regular healthcare-seeking behavior and PLCDs who visited hospitals with an escort suggest that having a friend or family member matters. Friends and family may offer psychosocial support to their loved ones which motivates them to take regularly seek healthcare services and to improve maintenance of their chronic conditions. To improve current efforts, providers may wish to examine the feasibility of incorporating family/friends into their patient’s healthcare plans. To ensure stronger linkages between provider, household, and PLCDs, policymakers may want to shift their focus towards primary hospitals and seek to ensure that primary hospitals are the managers of long-term treatment plans [39]. This shift can be enabled through strategic mass media campaigns at the community level so as to enhance public awareness of localized chronic disease maintenance [39,40,41]. Our study also found, consistent with other studies [14,42], that elderly participants sought health services more regularly than younger participants. This finding may come from two things: (1) the elderly may have more free time to seek care; and (2) the elderly may be more conscious of their healthcare needs [43]. Targeting elderly PLCDs may benefit this critical population, but it may also help younger PLCDs who may seek advice and encouragement from elderly PLCD to take on a more proactive role in the management of their chronic conditions.

Lastly, our study finds that economic factors like household income and economic burden of disease are significantly associated with regular health-seeking behavior by PLCD. There are several reasons that explain these findings. First, PLCD seek health services less where there is a reduction in household income [14,27]. Similarly, previous studies have reported that a higher economic burden of disease leads to a lower level of motivation to obtain regular care [35,40]. Moreover, many studies assume that decreased economic burden of disease results from insurance reimbursement, which will promote health service utilization. Existing health insurance compensation for PLCD remains low and in many parts of China, insurance reimbursement policy only focuses on the most severe chronic diseases, such as for diabetes with serious complications and hypertension at grade 3. The specific effect of insurance reimbursements was excluded in this study’s analyses, which differed from previous studies [9]. Moreover, maximum reimbursement for serious chronic diseases does not always cover long-term costs, resulting in high out-of-pocket costs that increase patients’ economic burden [44]. For example, the proportions of Hubei provincial government health expenditures, social health expenditures, and out-of-pocket payments in total health expenditures (THEs) were 24.62%, 28.66%, and 46.72%, respectively, in 2009. These numbers respectively increased to 30.02%, 30.32%, and 39.66% in 2011. Out-of-pocket payments still account for the largest proportion of THEs [45]. We thereby suggest that health plans need to be precisely designed so that they increase reimbursement levels for chronic diseases. We also underscore the need for supplementary health insurance that could specifically target PLCD at whatever stage of illness. 

## 5. Limitations

There were several limitations with our methods in this study. First, the two study areas were purposively selected in Hubei Province, a populous, landlocked province located in central China. Our selection of Hubei Province may reduce the generalizability of our findings because there may be socio-demographic factors that are unique to the province itself; secondly, the response rate was relatively low. This is largely due to the requirement that patients participate in periodic, data-gathering sessions (spanning 12 sessions over one year). This low response rate also invites a degree of selection bias. We, however, sought to minimize selection bias by following the recommendations of healthcare workers in sub-district and township facilities. We conducted surveys during weekends or after 5 pm during the workweek to avoid standard work hours. Our pursuit of a diverse sample size (e.g., employed, unemployed/retired) could have minimized the impact of selection bias in this study. 

## 6. Conclusions 

The rise of chronic diseases in middle-income countries like China has presented a critical threat to global efforts to curb healthcare costs and improve population health. Diabetes and hypertension are among the most prevalent chronic diseases in China and yet little is known about the individual and social factors that are associated with positive healthcare-seeking behaviors and regular health maintenance. More specifically, few studies, to date, have examined the individual/social factors that are associated with regular care and treatment for diabetes and hypertension [14]. This study addresses the dearth in existing literature and finds that social factors (i.e., usual source of care, social support from family or friends), household economic factors (i.e., household income, economic burden of disease), and individual factors (i.e., comorbidity, age) are significantly associated with a regular utilization of care/treatment services. 

The study posits that improvements in health maintenance for people living with diabetes and hypertension could be achieved through four approaches. First, primary hospitals could seek to ensure that critical services are available and remain affordable for people living with chronic diseases like diabetes and hypertension. These hospitals could also be linked together via strengthened health information systems, which can ensure accurate and timely monitoring of PLCDs; Secondly, because PLCDs commonly self-refer to higher-tiered hospitals, it important to encourage vertical integration of health information systems between primary-, secondary-, and tertiary- level hospitals; Thirdly, our examination has shown that support networks matter in efforts to ensure regular maintenance of chronic diseases. Family and friends should thereby be integrated into treatment plans. These treatment plans could be established and managed at primary hospitals, which could be better suited to provide patient-centered treatment plans than higher-tiered hospitals. Lastly, reimbursement rates for treatment/care for chronic conditions is markedly low. Increasing these rates could incentivize the provision of high-quality healthcare services. Similarly, a supplementary health insurance scheme could help address the healthcare needs of PLCDs, namely diabetic and/or hypertensive patients.

## Figures and Tables

**Table 1:** Socio-demographic characteristics of the patients with diabetes or hypertension.

Characteristics	Participants (*N* = 510)	Percent (%)
Gender		
Male	251	49.2
Female	259	50.8
Age		
<50	62	12.1
50–59	109	21.4
60–69	155	30.4
≥70	184	36.1
Education level		
No formal education	92	18.0
1–6 years study	155	30.4
7–9 years study	150	29.4
>9 years study	113	22.2
Marital status		
Unmarried	11	2.1
Married	422	82.8
Divorced	11	2.2
Widowed	66	12.9
Chronic disease		
Hypertension	363	71.18
Diabetes	73	14.31
Hypertension and Diabetes	74	14.51
Type of health insurance		
Medical insurance for urban works	234	45.9
Medical insurance for resident or NRCM ^1^	276	54.1
Household income (CNY) ^2^		
Low ^3^	209	40.9
Fair ^4^	127	25.0
Good ^5^	174	34.1
Household members		
1	31	6
≥2	479	94
Severity of chronic disease		
Grade 1 ^6^	186	36.5
Grade 2 ^7^	203	39.8
Grade 3 ^8^	121	23.7
Place of residence		
Rural	250	49.0
Urban	260	51.0
Assessment of Health-Seeking Behavior		
Regular	215	42.2
Irregular	295	57.8

Note: ^1^ NRCM: New Rural Co-operative Medical System; ^2^ Household income: urban household disposable income and rural household net income. The economic grade categories standard were based on 2015 criteria cited in the Hubei Statistical Yearbook; ^3^ Low: less than CNY 20,000 (3211 USD) per year in rural area; less than CNY 30,000 (4817 USD) per year in urban area; ^4^ Fair: CNY 20,000 (3211 USD) to CNY 40,000 (6422 USD) per year in rural area; CNY 30,000 (4817 USD)–60,000 (9633 USD) in urban area; ^5^ Good: CNY 40,000 or above per year in rural area; CNY 60,000 (9633 USD) or above per year in urban area; ^6^ Grade 1: the mild level condition; ^7^ Grade 2: the moderate level condition; ^8^ Grade 3: the severe level condition.

**Table 2:** Bivariate correlation of regularity of patients with hypertension or diabetes seeking health services.

	Regular ^1^ *N* (%)	Irregular ^2^ *N* (%)	*p*-Value
Individual factors	215 (42.2)	295 (57.8)	
Age			
<50	15 (7.0)	47 (15.9)	0.001
50–59	38 (17.7)	71 (24.1)	
60–69	71 (33.0)	84 (28.5)	
≥70	91 (42.3)	93 (31.5)	
Education level			
No formal education	35 (16.3)	57 (19.3)	0.065
1–6 years study	62 (28.8)	93 (31.5)	
7–9 years study	58 (27.0)	92 (31.2)	
>9 years study	60 (27.9)	53 (18.0)	
Marital status			
Unmarried	5 (2.3)	6 (2.0)	0.412
Married	171 (79.5)	251 (82.7)	
Divorced	6 (2.8)	5 (2.2)	
Widowed	33 (15.3)	33 (12.9)	
Comorbidity ^3^			
1 sort	86 (40.0)	187 (63.4)	<0.001
2 sorts	108 (50.2)	87 (29.5)	
≥3 sorts	21 (9.8)	21 (7.1)	
Self-reported health score			
Low (<60)	24 (11.2)	51 (17.2)	0.091
Medium (60–80)	105 (48.8)	122 (41.4)	
High (>80)	86 (40.0)	122 (41.4)	
Economic factors			
Economic burden of disease			
High ^4^	42 (19.5)	163 (55.3)	<0.001
Normal	173 (80.5)	132 (44.7)	
Health insurance			
Medical insurance for urban works	117 (54.4)	117 (39.7)	0.001
Medical insurance for residents or NRCM	98 (45.6)	178 (60.3)	
Household income (CNY) ^5^			
Low ^6^	56 (26.0)	153 (51.9)	<0.001
Fair ^7^	69 (32.1)	58 (19.7)	
Good ^8^	90 (41.9)	84 (28.5)	
Social factors			
Head of household			
No	96 (44.7)	129 (43.7)	0.836
Yes	119 (55.3)	166 (56.3)	
Number of household members with chronic diseases			
1	80 (37.2)	130 (44.1)	0.072
≥2	135 (62.8)	165 (55.9)	
Areas of residence			
Rural	127 (59.1)	123 (41.7)	<0.001
Urban	88 (40.9)	172 (58.3)	
The economic contribution of the family			
Major ^9^	121 (56.3)	147 (49.8)	0.301
Minor ^10^	66 (30.7)	109 (36.9)	
No ^11^	28 (13.0)	39 (13.3)	
Social Support (escort/someone to accompany to hospital visits)			
No	87 (40.5)	48 (16.3)	<0.001
Yes ^12^	128 (59.5)	247 (83.7)	
Usual provider			
Others ^13^	162 (75.3)	114 (38.6)	<0.001
Usual primary care provider ^14^	53 (24.7)	181 (61.4)	

Notes: ^1^ Regular: different grades patients seeking health services each month, 2 months, and 3 months a time or more respectively regularly in 2015; ^2^ Irregular: different grades patient seeking health services below each month, 2 months, and 3 months a time respectively in 2015; ^3^ Comorbidity: 1 sort-no comorbidity; 2 sort-1 comorbidities; 3 sort-2 comorbidities; ^4^ “Heavy” means catastrophic health expenditure per household, exceeds 40 percent of the disposable household income in past year. “Normal” means that there were no catastrophic health expenditures; ^5–8^ see notes 2–5 in Table 1; ^9^ “Major contribution” means family member’s income is above the income of other family members; ^10^ “Minor contribution” means family member’s income is less than 50 percent of the household income; ^11^ No contribution means family member has no income; ^12^ Hospital accompaniment means that the people living with chronic conditions (PLCD) sought care with the support of a family member or a friend; ^13^ Others: high-level hospitals; ^14^ Usual provider was determined by asking whether the PLCD had a usual primary care provider.

**Table 3:** Results of multivariate logistic regression analyses examining factors associated with regular clinical visits among hypertension and diabetes patients.

Predictors	Adjusted			Unadjusted		
	B ^1^	OR ^2^	95% CI ^3^	B	OR	95% CI
Age (ref < 50)						
50–59	0.891	2.437	0.996–5.964	0.517	1.677	0.831–3.384
60–69	0.867 *	2.379	1.026–5.514	0.974 **	2.648	1.367–5.132
≥70	1.325 **	3.762	1.617–8.751	1.12 **	3.066	1.602–5.868
Comorbidity (ref = 1 sorts)						
2 sorts	0.943 **	2.567	1.512–4.359	0.993 **	2.699	1.845–3.950
≥2 sorts	1.498 **	4.473	1.843–10.855	0.777 *	2.174	1.128–4.193
Economic burden of disease (ref = High)						
Normal	1.725 **	5.611	3.273–9.618	1.627 **	5.086	3.383–7.648
Household income (CNY) ^4^ (ref = Low ^5^)						
Fair ^6^	1.797 **	6.031	3.153–11.535	1.179 **	3.25	2.043–5.171
Good ^7^	1.808 **	6.097	3.341–11.126	1.074 **	2.927	1.910–4.485
Place of residence (ref = rural)						
Urban	0.87 **	2.387	1.352–4.212	0.702 **	2.018	1.412–2.884
Hospital accompaniment (ref = No)						
Yes	1.598 **	4.945	2.813–8.691	1.252 **	3.498	2.317–5.281
Usual provider (ref = Primary hospitals)						
Others	2.13 **	8.418	4.812–14.727	1.58 **	4.853	3.290–7.159

Note: ^1^ B: regression coefficient; ^2^ OR: odds ratio; ^3^ CI: confidence interval; Only significant correlates are presented. * *p* < 0.05, ** *p* < 0.01. The unadjusted models were conducted by univariate regression analysis; ^4–7^ See notes 5–8 in Table 1.
